# Nrf2-Mediated Ferroptosis Inhibition Exerts a Protective Effect on Acute-on-Chronic Liver Failure

**DOI:** 10.1155/2022/4505513

**Published:** 2022-04-16

**Authors:** Jing Wu, Ran Xue, Muchen Wu, Xuehong Yin, Bangxiang Xie, Qinghua Meng

**Affiliations:** ^1^Department of Liver Disease, Beijing You-An Hospital, Capital Medical University, Beijing 100069, China; ^2^Key Laboratory of Carcinogenesis and Translational Research (Ministry of Education), Department of Gastrointestinal Oncology, Peking University Cancer Hospital & Institute, Beijing 100036, China; ^3^Beijing Youan Hospital, Beijing Institute of Hepatology, Capital Medical University, Beijing 100069, China

## Abstract

Although massive hepatocyte cell death and oxidative stress constitute major events of acute-on-chronic liver failure (ACLF), the relationship of ferroptosis with ACLF has yet to be explored. Nuclear factor erythroid 2-related factor 2 (Nrf2) is a key regulator of ferroptosis. However, if Nrf2 modulates ACLF through ferroptosis remains unknown. Here, the liver tissues of ACLF patients were collected and murine models of ACLF using carbon tetrachloride, D-galactosamine, and lipopolysaccharide as well as an H_2_O_2_-induced hepatocyte injury model were established. Upon ACLF, livers exhibited key features of ferroptosis, including lipid peroxidation (increase in malondialdehyde whereas a decrease in glutathione and nicotinamide adenine dinucleotide phosphate), and increased mRNA expression of prostaglandin-endoperoxide synthase-2 (PTGS2). Ferroptosis inducer RSL-3 treatment aggravated liver damage, while ferroptosis inhibitor Ferrostatin-1 administration alleviated ACLF severity, manifesting with improved liver histopathological lesions and reduced serum ALT and AST. Compared with normal liver tissue, Nrf2 was upregulated in ACLF patients and murine models. Pharmacological activation of Nrf2 (Bardoxolone Methyl) attenuated liver damage, prevented lipid peroxidation, upregulated PTGS2 mRNA expression, and improved ferroptosis-specific mitochondrial morphology in vivo. In contrast, Nrf2 inhibitor ML385 exacerbated lipid peroxidation and liver injury. Collectively, Nrf2 plays a protective role in ACLF progression through repressing ferroptosis, which provides promising therapeutic cues for ACLF.

## 1. Introduction

Acute-on-chronic liver failure (ACLF) is a distinct clinical entity when chronic liver disease undergoes acute insults. ACLF is complicated with organ failures, characterized by rapid deteriorated course and high short-term mortality [1]. Globally, it is estimated that 24% to 40% of patients with cirrhosis admitted to hospitals were diagnosed with ACLF [2]. Deciphering ACLF pathogenesis and developing therapeutic strategies have become unmet needs and critical priority. Nevertheless, molecular mechanisms of progressive liver failure have hitherto not been fully understood, and ACLF remains one of the most challenging problems in clinic. Therefore, intense research efforts to delay disease progression are urgently required.

Hepatic cell death is a crucial molecular event of ACLF. Although apoptosis [3], autophagy [4], and necrosis [5] have been proposed in ACLF, whether other types of cell death are pathophysiological mechanisms underlying ACLF has not been explored. Ferroptosis is a novel mode of iron-dependent cell death manifesting with overwhelming lipid peroxidation and loss of cellular redox homeostasis [6]. Ferroptosis is morphologically, genetically, and biochemically distinct from other types of cell death [7, 8]. Accumulating evidence suggests that ferroptosis plays an unneglectable role in regulating disease development and progression, including neoplastic [9], neurological [10], and heart diseases [11]. Given that the liver is highly predisposed to oxidative damage and iron accumulation has been involved in multiple liver diseases [12], ferroptosis is a potential contributor to various liver diseases. Dysregulated iron homeostasis has been reported in patients with ACLF [13]. Specifically, increased circulating levels of total iron and ferritin were observed in ACLF patients relative to normal controls. Since aberrant iron metabolism is a potential predictor of multiorgan failure and mortality in patients with ACLF [13, 14], we hypothesized that ferroptosis contributed to ACLF pathogenesis. To the best of our knowledge, there has been no clear demonstration of association between ferroptosis and ACLF.

The nuclear factor erythroid 2-related factor 2 (Nrf2) is a vital nuclear transcription factor, which controls a battery of cellular defensive genes to maintain redox homeostasis and cell survival [15]. Recently, Nrf2 has been identified as a regulator of ferroptosis. For example, in neoplastic diseases, Nrf2-mediated ferroptosis suppressed tumor growth and sensitized cancer cells to antitumor drugs [16, 17]. In acute or chronic tissue/cell damage, Nrf2 stabilization could restrain ferroptosis and subsequently relieve injury [18]. Specifically, activation of Keap1/Nrf2-ARE signaling pathway in response to dehydroabietic acid could eliminate reactive oxygen species (ROS) accumulation and suppress ferroptosis, which consequently, improved nonalcoholic fatty liver disease [19]. However, little is known whether ferroptosis is a mechanism through which Nrf2 confers a protective effect on ACLF.

The purpose of this study was to investigate if ferroptosis participates in ACLF pathogenesis and to unveil underlying molecular mechanisms. Major features of clinical ACLF were recapitulated through establishing murine models with carbon tetrachloride (CCl4), D-galactosamine (D-gal), and lipopolysaccharide (LPS). A hepatocyte injury model was established by treating L02 cells with H_2_O_2_ in vitro. Successful establishment of ACLF model was confirmed by features of ACLF in terms of laboratory parameters and liver histopathology. Then, mice were treated with either ferroptosis inducer or inhibitor to assess effects of ferroptosis on liver injury. Finally, we explored if Nrf2 could protect from liver injury through ferroptosis by using either inducer or inhibitor of Nrf2.

## 2. Materials and Methods

This study was conducted following the Declaration of Helsinki. All animal experiments were performed according to the guidelines of Animal Experiments and Experimental Animal Welfare Committee at Capital Medical University. This study was approved by the Ethical Committee of Beijing You-An Hospital, Capital Medical University (No. AEEI-2020-195).

### 2.1. Mice and Experimental Design

Male BALB/c mice between 6 and 8 weeks of age were purchased from the Beijing Weitong Lihua Experimental Animal Ltd. Co. (license: SCXK11-00-0008). Mice were housed in a standard environmental condition with sterile water and diet, the 23°C laboratory room temperature, 12 h of light and 12 h of darkness, and 50% indoor humidity were acclimated for two weeks before experimentation. ACLF models were constructed as follows: mice were injected intraperitoneally (i.p.) with 0.2 ml CCl4 in olive oil (CCl4/olive oil volume = 1 : 5, twice a week) for 8 weeks, and subsequently with a high frequency (three times a week) for 4 weeks. Twenty-four hours later, mice were challenged i.p. with LPS (10 *μ*g/kg) and D-gal (500 mg/kg).

**Figure 1:**
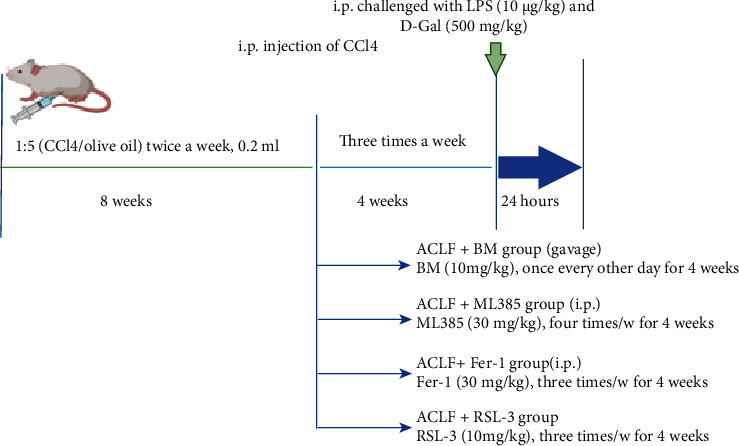
The experimental regimen applied in this study.

Mice were randomly divided into six groups. Group 1 (*n* = 3), normal control: mice received 0.2 ml of saline (0.9%, v/v) in water (twice a week for 8 weeks and 3 times a week for 4 weeks). Group 2, ACLF (*n* = 6): ACLF models were constructed as described above. Group 3 (*n* = 6), ACLF + ferroptosis activation: mice were administrated i.p. with ferroptosis inducer RSL-3 (10 mg/kg, Abmole, USA) three times a week for 4 weeks. Group 4 (*n* = 6), ACLF + ML385: mice were injected i.p. with Nrf2 inhibitor ML385 (30 mg/kg, Abmole, USA) four times per week for 4 weeks. Group 5 (*n* = 6), ACLF + Baroxolone Methyl (BM): mice were administrated with BM (10 mg/kg, dissolved in olive oil, Abmole, USA) by gavage once every other day for 4 weeks. Group 6 (*n* = 6), ACLF + Ferrostatin-1 (Fer-1): mice were treated with i.p. injection of Fer-1(10 mg/kg, Abmole, USA) three times a week for 4 weeks. The experimental regimen was described in Figure 1.

### 2.2. Patients

Between January 2019 and June 2021, five transplant recipients who fulfilled diagnostic criteria of Asian Pacific Association for the Study of the Liver (APASL) [20] for ACLF were included in this study. Normal liver tissues were obtained from liver transplant donors, serving as the healthy control. Exclusion criteria included multiple organ failure, fulminant hepatic failure, complicated liver cancer, long-term immunosuppressive therapy, and age less than 18 years. Informed consent was obtained from each participant before enrollment. This study was approved by the Ethical Committee of Beijing You-An Hospital, Capital Medical University (No. LL-2018-119-K).

### 2.3. Western Blot

The cells and liver tissues were lysed in lysis buffer and centrifuged at 4°C and 12000 rpm for 30 min. Nuclear proteins were extracted with the Nuclear Protein Extraction Kit (Solarbio, China). After assessment of concentrations, proteins were denatured using 5× sodium dodecyl sulfate (SDS) loading buffer at 100°C for 5 min. Protein mixtures were separated on 8-12% SDS-polyacrylamide gel electrophoresis and transferred to polyvinylidene difluoride membrane. After blocked with 5% defat milk at room temperature for 1 h, the membrane was incubated with the primary antibody against Nrf2 (Cell Signaling Technology, MA, USA), HO-1(Abcam, Cambridge, UK), and NQO1(Abcam, Cambridge, UK) overnight at 4°C. The next day, after washed for three times with Tris Buffered Saline with Tween 20 (TBST), the membrane was incubated with goat antirabbit horseradish peroxidase-conjugated secondary antibodies (Cell Signaling Technology, MA, USA) for 1 h at room temperature and washed for three times with TBST. Subsequently, the bands were visualized using an enhanced chemiluminescence detection kit (Thermo Fisher Scientific, USA) according to the manufacturer's instructions.

### 2.4. Liver Histopathology and Immunohistochemical Assays

As previously described [21], the liver tissues collected from different groups were fixed with formaldehyde and embedded in paraffin. Haematoxylin–eosin and Masson's trichrome staining were conducted to evaluate liver histological features and tissue fibrosis. Formalin-fixed paraffin-embedded liver tissues were stained with antibodies against Nrf2 (Sigma-Aldrich, St. Louis, MO, USA). The morphology was assessed under an electron microscope (Nikon Eclipse 80i, Tokyo, Japan). Representative pictures of liver sections from all groups were displayed.

### 2.5. Enzyme-Linked Immunosorbent Assay (ELISA)

ELISA kits (RayBiotech, Norcross, GA) were applied to detect hepatic protein levels of IL-6 and tumor necrosis factor (TNF)-*α* according to the manufacturer's protocol.

### 2.6. Serum Biochemistry

Serum alanine aminotransferase (ALT) and aspartate aminotransferase (AST) were detected by an automated chemical analyzer (Olympus Company, Tokyo, Japan).

### 2.7. Hepatic Content of Malondialdehyde (MDA) and Glutathione (GSH)

According to the manufacturer's recommendations, MDA and GSH levels were measured using corresponding detection kits (Beyotime, Beijing, China). Absorbance values of samples were measured at 532 nm and 412 nm, respectively.

### 2.8. Iron Assay

Hepatic iron concentration was determined by Iron Assay Kit (No. ab83366, Abcam) according to the manufacturer's instruction.

### 2.9. Cell Culture

Human cell line L02 was cultured in Dulbecco's Modified Eagle's Medium (DMEM) (Gibco, Gaithersburg, MD, USA) containing 10% fetal bovine serum (FBS) (Gibco, Gaithersburg, MD, USA) and grown at 37°C and 5% CO_2_ humidified atmosphere. Cells were treated with H_2_O_2_ (300 *μ*M) (Invitrogen, Carlsbad, CA, USA) for 30 h for hepatocyte injury model. Moreover, cells were treated with or without inducer or inhibitor of ferroptosis or Nrf2 for mechanistic exploration.

### 2.10. Cell Viability Assay

Cell Counting Kit-8 (CCK-8, Abmole, USA) was used to assess proliferation of cells with different treatments. Briefly, 3 × 10^3^ L02 cells were seeded in 96-well plates and incubated for 24 h. Afterwards, cells were pretreated with BM (0.2 *μ*M), Fer-1(0.25 *μ*M), and ML385 (10 *μ*M) for 12 h, respectively, and H_2_O_2_ (300 *μ*M) for 30 h. For the positive control group, cells were treated with Erastin (E, 10 *μ*M, an inducer of ferroptosis) for 30 h. Later, 10 *μ*L CCK-8 working solution was added to corresponding culture medium and incubated for 2 h at 37°C. Finally, absorbance was evaluated at 450 nm using a microplate reader.

### 2.11. Lipid Peroxidation Assay

Indicated cells were stained with 5 *μ*M BODIPY® 581/591 C11 dye for 30 min at 37°C in the dark. After the incubation, cells were washed twice with phosphate buffer saline (PBS) and resuspended in 400 *μ*L PBS. Flow cytometry analysis was conducted using a BD FACSCalibur system.

### 2.12. Transmission Electron Microscopy (TEM)

Liver tissues were fixed with glutaraldehyde (2.5%) and washed for three times with phosphate-buffered solution. After embedded in 1% agarose, samples were dehydrated in 30%, 50%, 70%, 80%, and 95% ethanol for 20 min, in Acetone for 15 min, and then embedded in Acetone. Resin blocks were cut to 60-80 nm-thin fragments on ultramicrotome. The tissue was fished out onto cuprum grids. After staining, images were taken under TEM (JEM-1200; Jeol Ltd.) at 80 kV with representative pictures being depicted.

### 2.13. Quantitative Real-Time Polymerase Chain Reaction (qRT-PCR)

TRIzol reagent (Invitrogen, Carlsbad, CA, USA) was used for total RNA isolation according to the manufacturer's instructions. Isolated RNA was reverse transcribed into cDNA using the PrimeScript RT reagent kit (TaKaRa Biotechnology, Beijing, China). Finally, real-time PCR was performed using the TB Green Premix Ex Taq™ (Tli RNaseH Plus) kit (TaKaRa Biotechnology, Beijing, China) on an ABI ViiA 7 Real-Time PCR System (ABI, USA) according to the manufacturer's protocol. The relative gene expression was normalized to glyceraldehyde-3-phosphate dehydrogenase (GAPDH). The thermal cycling conditions were as follows: 95°C for 30 s; 40 cycles of 95°C for 5 s and 60°C for 30 s; and dissociation at 95°C for 15 s, 60°C for 60 s, and 95°C for 15 s. Primers used in this study were listed in Table 1.

### 2.14. Statistical Analysis

The GraphPad Prism 8.0 software was used to conduct statistical analysis. Data were expressed as means ± standard deviation (SD). Unpaired Student's *t*-test and Mann–Whitney *U* test were used to evaluate the differences between groups. A *p* value < 0.05 was considered statistically significant.

## 3. Results

### 3.1. Induction of Ferroptosis Aggravated Liver Injury in ACLF Murine Models

**Figure 2:**
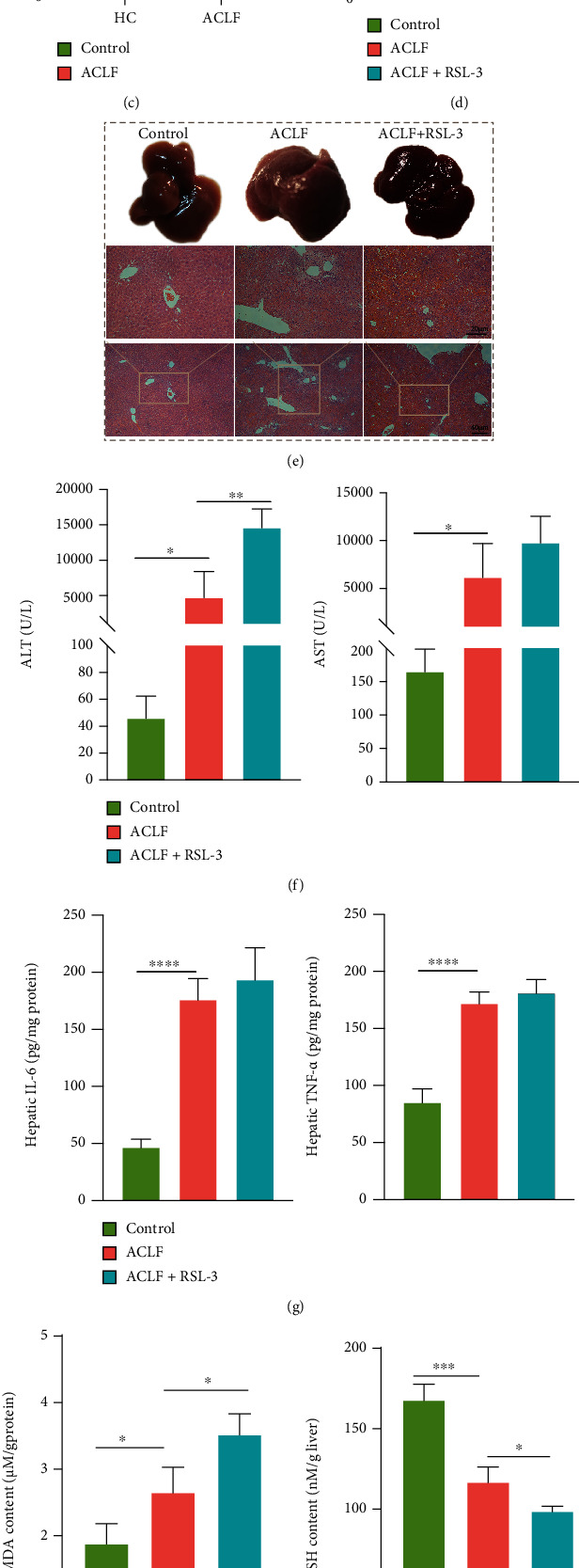
Ferroptosis aggravated liver injury in ACLF mice models. (a) Hepatic iron content both in healthy controls and ACLF patients was measured (HC *n* = 3, ACLF *n* = 5). (b) Hepatic NADPH content was decreased in ACLF patients relative to healthy controls (HC *n* = 3, ACLF *n* = 5). (c) PTGS2 mRNA expression was increased in patients with ACLF compared with healthy controls (HC *n* = 3, ACLF *n* = 5). (d) The livers of the ACLF+RSL-3-treated mice were slightly heavier than those of ACLF mice although without statistical significance. (e) Representative images of morphologic and histopathological features of the control, ACLF, and ACLF+RSL-3-treated mice. Original magnification ×100 (Bar = 40 *μ*m) and ×200 (Bar = 20 *μ*m). (f and g) Serum ALT and AST levels and hepatic inflammatory cytokines (IL-6, TNF-*α*) were assessed. (h–j) Lipid peroxidation was analyzed through comparing hepatic GSH, NADPH, and MDA in the three groups. (k) The expression of PTGS2 mRNA was measured by qRT-PCR. Data are expressed as mean ± SD^∗^*p* < 0.05, ^∗∗^*p* < 0.01, ^∗∗∗^*p* < 0.001, ^∗∗∗∗^*p* < 0.0001. *n* = 3 (control, ACLF+RSL-3), *n* = 6 (ACLF). ALT = alanine aminotransferase; AST = aspartate aminotransferase; ACLF = acute-on-chronic liver failure; GSH = glutathione; IL-6 = interleukin-6; MDA = malondialdehyde; NADPH = nicotinamide adenine dinucleotide phosphate; PTGS2 = prostaglandin-endoperoxide synthase-2; qRT-PCR = quantitative real-time polymerase chain reaction; TNF-*α* = tumor necrosis factor alpha.

Human hepatic iron concentration was evaluated in the healthy controls and ACLF patients. Compared with the healthy controls, hepatic iron content was significantly increased in ACLF (Figure 2(a)), consistent with previous studies [13]. Decreased hepatic nicotinamide adenine dinucleotide phosphate (NADPH) content, an established signature of ferroptosis [22, 23], was observed in ACLF relative to the healthy controls (Figure 2(b)). In addition, the mRNA expression of prostaglandin-endoperoxide synthase-2 (PTGS2), another typical feature of ferroptosis [22], was elevated in ACLF (Figure 2(c)). We speculated that ferroptosis was implicated in ACLF pathogenesis.

To verify this hypothesis, a mouse model was established to recapitulate major characteristics of clinical ACLF using CCl4, LPS, and D-gal (Figure 1). Induction of ferroptosis, through treatment with RSL-3, an inducer of ferroptosis [24], reinforced liver damage. As shown in Figures 2(d) and 2(e), in the control group, the livers were smooth and rosy with intact hepatic structure; while in the ACLF group, the livers were smaller and harder, with blunt edges and small nodules. Disordered hepatic lobule structure, substantial hepatic cell death, and advanced fibrosis with nodule formation, as major characteristics of ACLF [1], were observed in the successfully established ACLF group. Of note, these histopathological lesions were more evident in the livers of RSL-3-treated mice. Consistent with an increase in histopathological severity, serum biochemical indicators (ALT and AST) were elevated in response to RSL-3 treatment (Figure 2(f)). Regarding inflammatory cytokines, however, no significantly increased hepatic IL-6 and TNF-*α* were observed in response to RSL-3 treatment compared with the ACLF group (Figure 2(g)). In parallel with aggravated liver damage, several indicators of lipid peroxidation, (a) MDA (Figure 2(h)), an end product of lipid peroxidation, was higher in the ACLF group and the highest in the RSL-3 treatment group; (b) GSH and NADPH showed an opposite trend (Figures 2(i) and 2(j)). Similarly, the mRNA expression of PTGS2 was elevated along with increased severity of liver injury in the ACLF and RSL-3 groups (Figure 2(k)). Collectively, ferroptosis might act as a deleterious factor, which aggravated liver damage and promoted disease progression in ACLF.

### 3.2. Activation of Nrf2 Inhibited Ferroptosis and Ameliorated Liver Injury In Vivo

**Figure 3:**
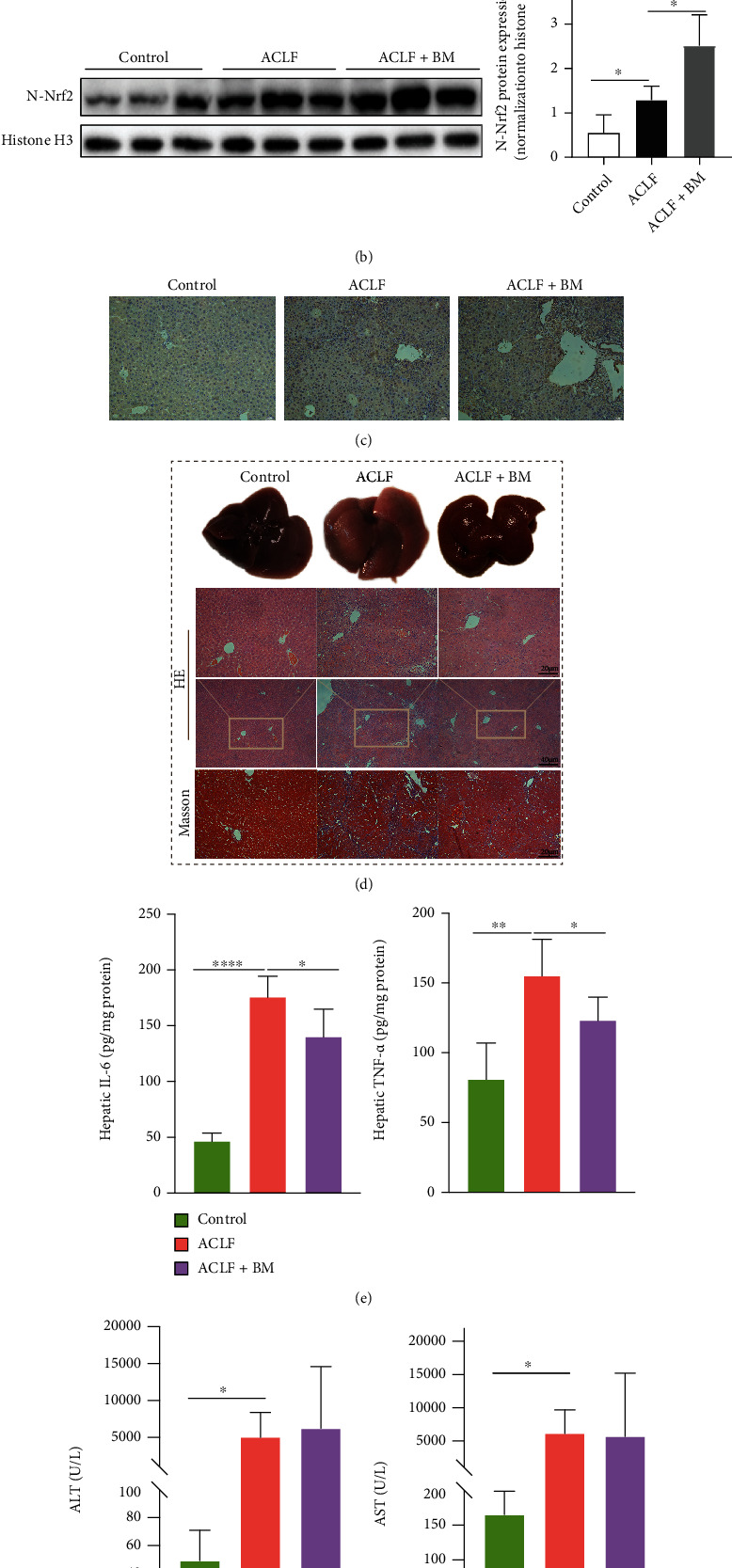
Activation of Nrf2 ameliorated liver injury in vivo. (a and b) The protein expression of Nrf2 in the liver tissues was confirmed by western blot (HC *n* = 3, ACLF *n* = 5). (c) The results of immunohistochemical staining indicated that Nrf2 translocated into the nucleus and activated in the pathologic context of ACLF, and BM treatment further augmented its activation. Original magnification ×200 (Bar = 20 *μ*m). (d) Improvement of morphologic and histopathological features in the BM treatment group implied the protective effect of Nrf2 on ACLF. Original magnification ×100 (Bar = 40 *μ*m) and ×200 (Bar = 20 *μ*m). (e) Hepatic protein levels of IL-6 and TNF-*α* were measured. (f) BM treatment did not significantly decrease serum levels of ALT and AST. ^∗^*p* < 0.05, ^∗∗^*p* < 0.01, ^∗∗∗∗^*p* < 0.0001. *n* = 3 (control), *n* = 6 (ACLF), *n* = 5 (ACLF+BM). ALT = alanine aminotransferase; AST = aspartate aminotransferase; ACLF = acute-on-chronic liver failure; BM = bardoxolone methyl; IL-6 = interleukin-6; Nrf2 = nuclear factor erythroid 2-related factor 2; TNF-*α* = tumor necrosis factor alpha.

Nrf2-mediated defensive network might protect against various pathologic injuries and engage in regulating ferroptosis [25]. When being activated, Nrf2 was translocated to nuclear and initiated downstream anoxidative genes [26, 27]. Our preliminary results showed that nuclear protein expressions of Nrf2 were significantly increased in the liver tissues of ACLF patients compared with normal ones (Figure 3(a)). In addition, at mRNA levels, NAD(P) H quinone dehydrogenase, quinone 1 (NQO1), a pivotal target gene of Nrf2, was upregulated in ACLF liver tissues compared with normal ones (Supplemental Figure 1). Therefore, we speculated that Nrf2 might have a protective role in ferroptosis-provoked liver damage. BM is a common agent to activate Nrf2 [28, 29]. Nrf2 was activated in vivo in the BM group, as revealed by increased nuclear protein expression of Nrf2 relative to the ACLF group (Figure 3(b)). As expected, immunohistochemical staining of Nrf2 demonstrated the same results as western blots (Figure 3(c)). Specifically, in the control group, a small proportion of Nrf2 positive cells were diffusely distributed in the cytoplasm. In contrast, in the ACLF group, a large proportion of Nrf2 positive cells in the nuclei were identified. In the BM group, Nrf2 expressed in nuclei was largely augmented. Alongside Nrf2 activation was attenuated severity of ACLF, which was confirmed by gross morphological and histopathological features of the livers (Figure 3(d)). Specifically, Nrf2-activated livers demonstrated mitigated inflammation and hepatocytes death along with improved hepatic lobule structure disorder. In addition, this pattern was confirmed by decreased hepatic inflammatory indicators, such as IL-6 and TNF-*α* (Figure 3(e)). Contrary to our expectation, BM treatment failed to reduce the serum levels of ALT and AST (Figure 3(f)).

**Figure 4:**
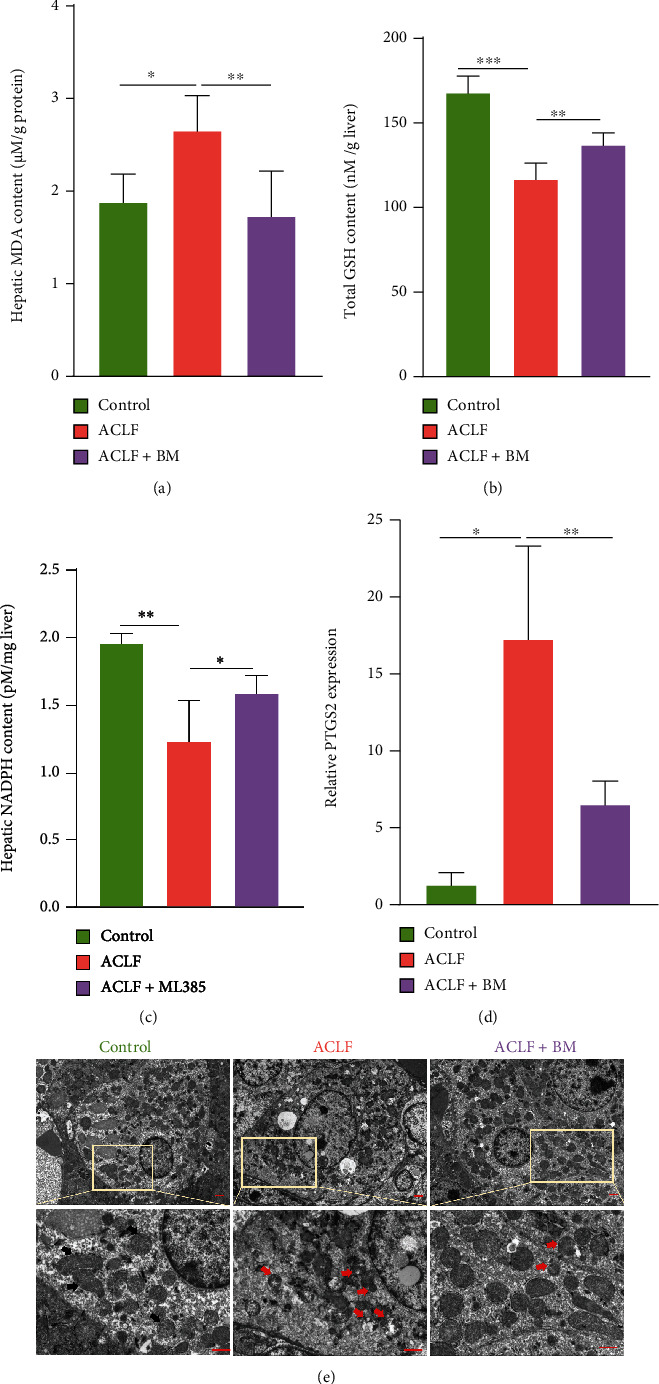
Activation of Nrf2 mitigated lipid peroxidation. (a) Hepatic MDA content was measured in all three groups. (b) Total GSH concentration was detected. (c) Hepatic NADPH content was measured. (d) The relative mRNA expression of ferroptosis-related gene PTGS2 in the liver tissues. (e) Increased mitochondrial outer membrane rupture and diminished mitochondrial ridges were seen in the ACLF group under electron microscopy, while BM treatment improved these morphological changes. Bar = 10 *μ*m. Black arrows indicate normal mitochondria; red arrows indicate shrunken and ruptured mitochondria. ^∗^*p* < 0.05, ^∗∗^*p* < 0.01, ^∗∗∗^*p* < 0.001. *n* = 3 (control), *n* = 6 (ACLF), *n* = 5 (ACLF+BM). ACLF = acute-on-chronic liver failure; BM = bardoxolone methyl; GSH = glutathione; MDA = malondialdehyde; NADPH = nicotinamide adenine dinucleotide phosphate; Nrf2 = nuclear factor erythroid 2-related factor 2; PTGS2 = prostaglandin-endoperoxide synthase-2.

Furthermore, the potential role of Nrf2 in ferroptosis during ACLF was examined. As shown in Figures 4(a)–4(c), BM increased hepatic content of GSH and NADPH whereas decreased content of MDA. In addition, PTGS2 was downregulated in response to BM treatment (Figure 4(d)). Apart from lipid peroxidation, mitochondrial morphology was examined. Compared to the control group, the liver tissues from the ACLF group displayed smaller mitochondria morphology with diminished mitochondria crista, as well as rupture of outer mitochondrial membrane, all of which were specific morphological features of ferroptosis [7], whereas BM treatment improved this morphological phenotype (Figure 4(e)). Accordingly, Nrf2 activation inhibited ferroptosis, which might hold a substantial potential in attenuating liver damage in ACLF.

### 3.3. Nrf2 Inhibition Promoted the Onset of Lipid Peroxidation and Corresponded to a More Severe Liver Injury

**Figure 5:**
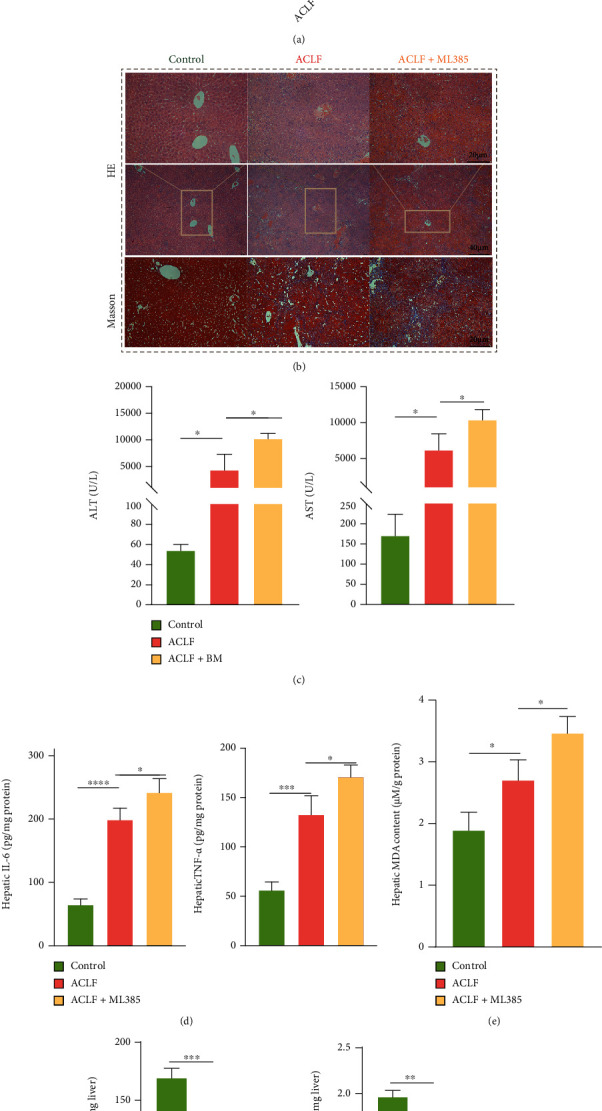
Nrf2 inhibition promoted the onset of lipid peroxidation and aggravated liver injury in mice. (a) Results of western blot confirmed the successful inhibition of Nrf2 using ML385 in vivo (*n* = 3). (b) The mice treated with ML385 demonstrated a more severe liver injury (hepatocytes necrosis, destruction of the lobular structure, infiltration of inflammatory cells, obvious vascular congestion, and hemorrhage) compared with the ACLF mice. Original magnification ×100 (Bar = 40 *μ*m) and ×200 (Bar = 20 *μ*m). (c) Significantly increased serum ALT and AST suggested the aggravated liver damage of mice treated with ML385. (d) ML385 treatment also increased the protein levels of inflammatory cytokines (IL-6, TNF-*α*). (e–g) The mice treated with ML385 showed increased lipid peroxidation as evidenced by decreased hepatic GSH and NADPH content and increased hepatic MDA levels. ^∗^*p* < 0.05, ^∗∗^*p* < 0.01, ^∗∗∗∗^*p* < 0.0001. *n* = 3 (control), *n* = 6 (ACLF), *n* = 3 (ACLF+ML385). ALT = alanine aminotransferase. AST = aspartate aminotransferase; ACLF = acute-on-chronic liver failure; GSH = glutathione; IL-6 = interleukin-6; MDA = malondialdehyde; Nrf2 = nuclear factor erythroid 2-related factor 2; NADPH = nicotinamide adenine dinucleotide phosphate; TNF-*α* = tumor necrosis factor alpha.

To verify that Nrf2 was required in improving lipid peroxidation and liver damage, ML385, an inhibitor of Nrf2 [30], was used to inactivate Nrf2. As demonstrated by western blot, nuclear content of Nrf2 was decreased, confirming inactivation of Nrf2 (Figure 5(a)). Consistent with decrease in Nrf2, more severe histopathologic lesions, such as hepatocytes necrosis, destruction of the lobular structure, infiltration of inflammatory cells, obvious vascular congestion, and hemorrhage and tissue fibrosis were observed in the ML385 group compared to the ACLF group (Figure 5(b)). Convergently, liver damage assessed by serum biochemical parameters including ALT and AST was exacerbated in the ML385-treated mice (Figure 5(c)). As Nrf2 inhibited inflammatory response, inflammatory factors including IL-6 and TNF-*α* were augmented in the Nrf2-inhibited livers (Figure 5(d)). As for oxidative stress, the liver tissue from ML385-treated mice demonstrated the highest content of MDA, whereas GSH and NADPH were markedly decreased compared with the ACLF group and controls (Figure 5(e)–5(g)). Taken together, targeting Nrf2 might hold a potential therapeutic value in treating ACLF.

### 3.4. Inhibiting Ferroptosis Attenuated the Severity of ACLF In Vivo

**Figure 6:**
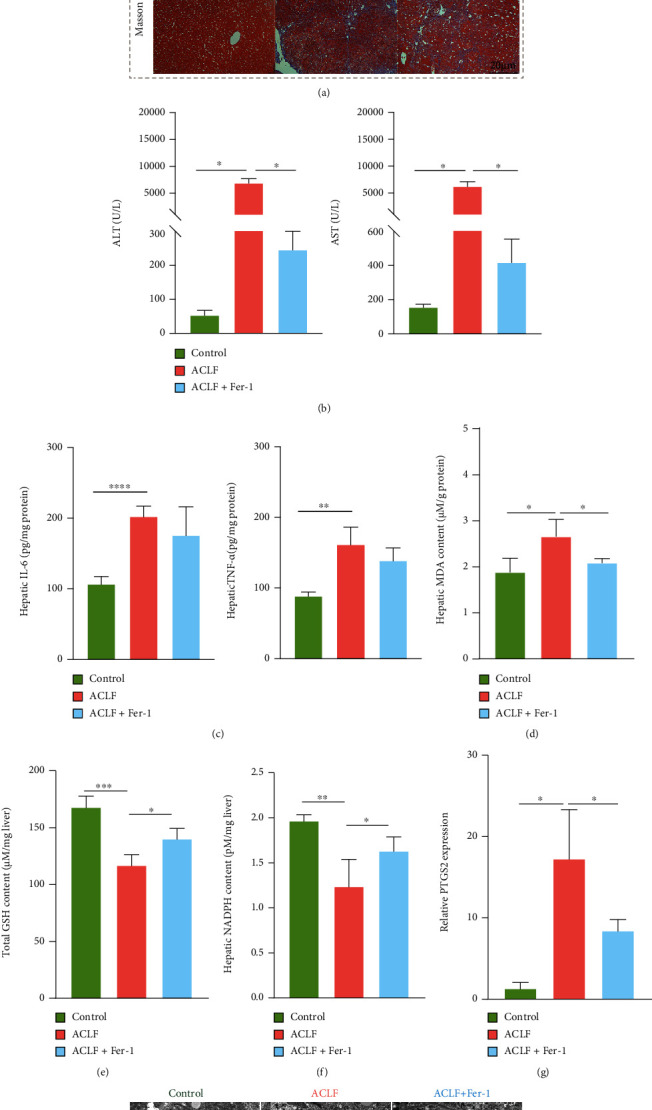
Repression of ferroptosis attenuates the severity of ACLF in vivo. (a) Representative images of morphological and histopathologic traits. Original magnification ×100 (Bar = 40 *μ*m) and ×200 (Bar = 20 *μ*m). (b) Fer-1 treatment significantly reduced the serum levels of ALT and AST. (c) Inhibition of ferroptosis failed to decrease the hepatic IL-6 and TNF-*α* concentrations. (d–f) Increased hepatic GSH and NADPH and decreased hepatic MDA were observed in mice treated with Fer-1. (g) The mRNA expression of ferroptosis-related gene PTGS2 was decreased in mice treated with Fer-1. (h) Fer-1 treatment improved ferroptosis-specific mitochondrial morphology. Bar = 10 *μ*m. Black arrows indicate normal mitochondria; red arrows indicate shrunken and ruptured mitochondria. ^∗^*p* < 0.05, ^∗∗^*p* < 0.01, ^∗∗∗^*p* < 0.001, ^∗∗∗∗^*p* < 0.0001. *n* = 3(control), *n* = 6 (ACLF), *n* = 5 (ACLF+Fer-1). ALT = alanine aminotransferase; AST = aspartate aminotransferase; ACLF = acute-on-chronic liver failure; Fer-1 = ferrostatin-1; GSH = glutathione; IL-6 = interleukin-6; MDA = malondialdehyde; NADPH = nicotinamide adenine dinucleotide phosphate; PTGS2 = prostaglandin-endoperoxide synthase-2; TNF-*α* = tumor necrosis factor alpha.

To confirm functions of ferroptosis in ACLF, ACLF mice were treated with ferroptosis-specific inhibitor Fer-1. Interestingly, Fer-1 ameliorated ACLF severity, manifesting with improved liver morphology and histopathologic lesions (e.g., reduced granules and improved lobule structure) (Figure 6(a)). Consistently, liver injury was revealed by critical indicators. Specifically, liver function indices (ALT and AST) in the Fer-1 treatment group were lower than that in the ACLF group, indicating a protective effect of Fer-1 on liver function (Figure 6(b)). Moreover, protein levels of hepatic inflammatory cytokines (IL-6 and TNF-*α*) were decreased in Fer-1-treated mice relative to the ACLF group (Figure 6(c)). Consistent with improved liver function, reduced lipid peroxidation was identified, evidenced by increased hepatic GSH and NADPH whereas decreased hepatic MDA in Fer-1-treated mice (Figures 6(d)–6(f)). Similarly, PTGS2 mRNA expression was reduced after Fer-1 treatment, indicating an improved lipid oxidative stress status (Figure 6(g)). In addition, TEM demonstrated improved ferroptosis-specific mitochondrial morphology after Fer-1 treatment (Figure 6(h)).

### 3.5. H_2_O_2_ Exposure Induced Ferroptosis in L02 Cells

**Figure 7:**
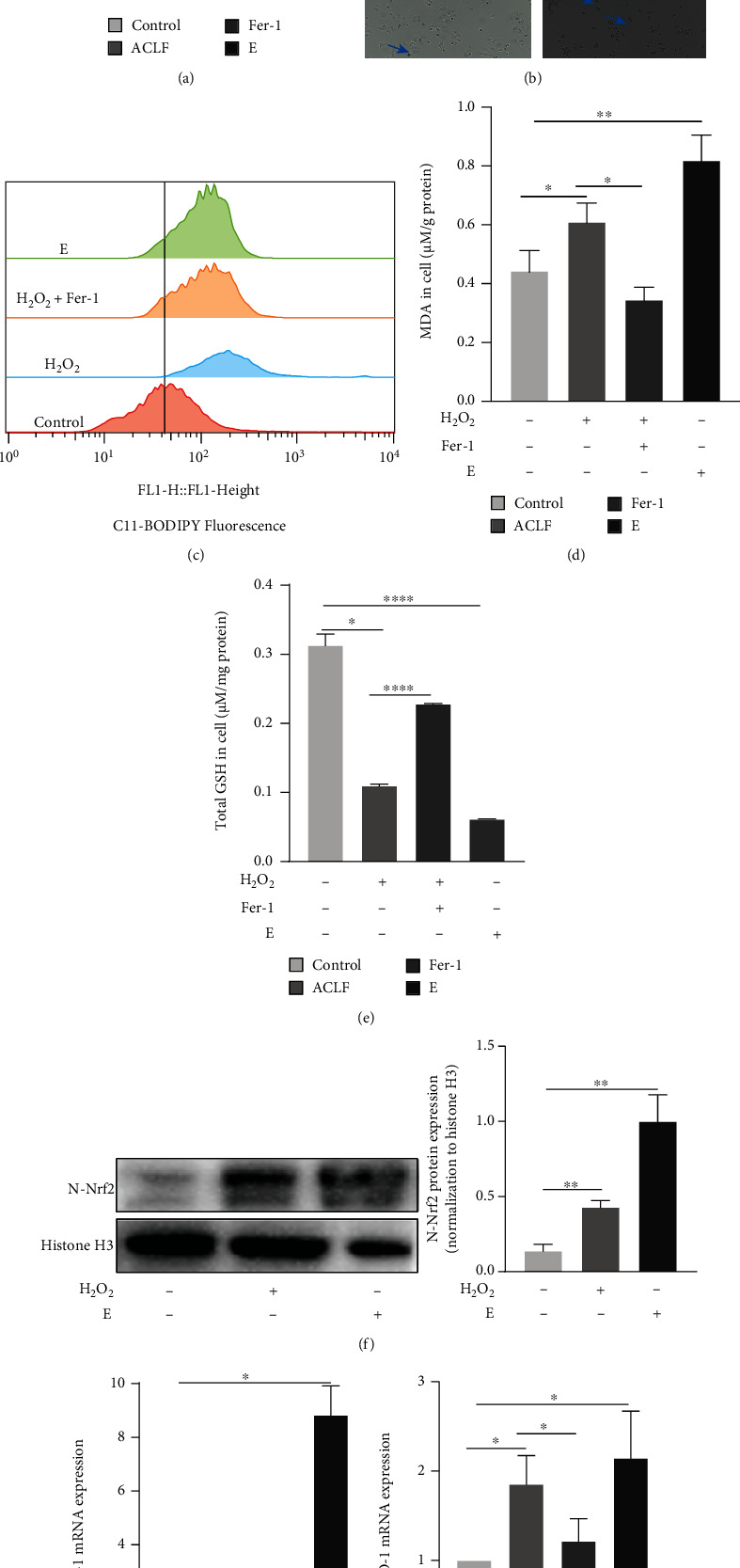
H_2_O_2_ exposure induced ferroptosis in L02 cells. (a) Relative cell viability was confirmed by the CCK-8 assay (*n* = 6). (b) L02 cells underwent ferroptosis-specific morphologic phenotype (cells shrunk at first and eventually exhibiting a balloon-type) after being treated with H_2_O_2_ or Erastin. The blue and red arrows indicate shrunken and balloon phenotypes, respectively ((a) normal L02 cells. (b) L02 cells treated with H_2_O_2_. (c) L02 cells co-treated with Fer-1 and H_2_O_2_. (d) L02 cells exposed to Erastin). Original magnification ×200. (c) Cells were stained with 5 *μ*M BODIPY® 581/591 C11 to detect the content of cellular lipid peroxide through flow cytometry (*n* = 3). (d and e) Cellular content MDA and total GSH were measured, respectively (*n* = 3). (f) Protein expression of Nrf2 was elevated after cells were treated with H_2_O_2_ or Erastin compared with normal L02 cells (*n* = 3). (g) The mRNA expression of HO-1 and NQO1 was elevated in response to H_2_O_2_ and Erastin treatment (*n* = 3). ^∗^*p* < 0.05, ^∗∗^*p* < 0.01, ^∗∗∗^*p* < 0.001. CCK-8 = Cell Counting Kit-8; Fer-1 = ferrostatin-1; GSH = glutathione; HO-1 = heme oxygenase-1; MDA = malondialdehyde; NQO1 = NAD(P) H quinone dehydrogenase, quinone 1.

To further explore engagement of ferroptosis in ACLF, a hepatocyte injury model was established via treating L02 cells with H_2_O_2_ for 30 h. Cells were divided into 4 groups as follows: control, ACLF, ACLF + Fer-1, and control + Erastin (E) (serving as the positive control). CCK-8 assay indicated that H_2_O_2_ inhibited cell viability, as exhibited in the control+E group, whereas Fer-1 reversed growth inhibition, as evidenced by improved viability in the ACLF+Fer-1 group (Figure 7(a)). L02 cells grew slowly and became skinnier, accompanied by decreased attachment following H_2_O_2_ and Erastin treatment. These cells eventually exhibited a “ballooning” phenotype because of plasma membrane destabilization, cytoskeletal rearrangements, and disruption of proteostasis [31]. By contrast, Fer-1 improved morphology (Figure 7(b)). H_2_O_2_ increased lipid ROS in a comparable pattern to Erastin treatment, which was counteracted by Fer-1 (Figure 7(c)). In parallel, compared with the control group, increased MDA whereas decreased GSH were observed in the H_2_O_2_ and Erastin treatment groups, which was rescued by Fer-1 (Figures 7(d) and 7(e)). Simultaneously, nuclear protein expression of Nrf2 was upregulated after H_2_O_2_ and Erastin treatment (Figure 7(f)). In addition, at protein (Supplementary Figure 2) and mRNA levels (Figure 7(g)), Nrf2 target genes heme oxygenase-1 (HO-1) and NQO1 were upregulated in response to H_2_O_2_ and Erastin treatment. Collectively, H_2_O_2_ treatment contributed to ferroptosis in L02 cells.

### 3.6. Nrf2 Protected against H_2_O_2_-Induced Hepatotoxicity via Inhibiting Ferroptosis In Vitro

**Figure 8:**
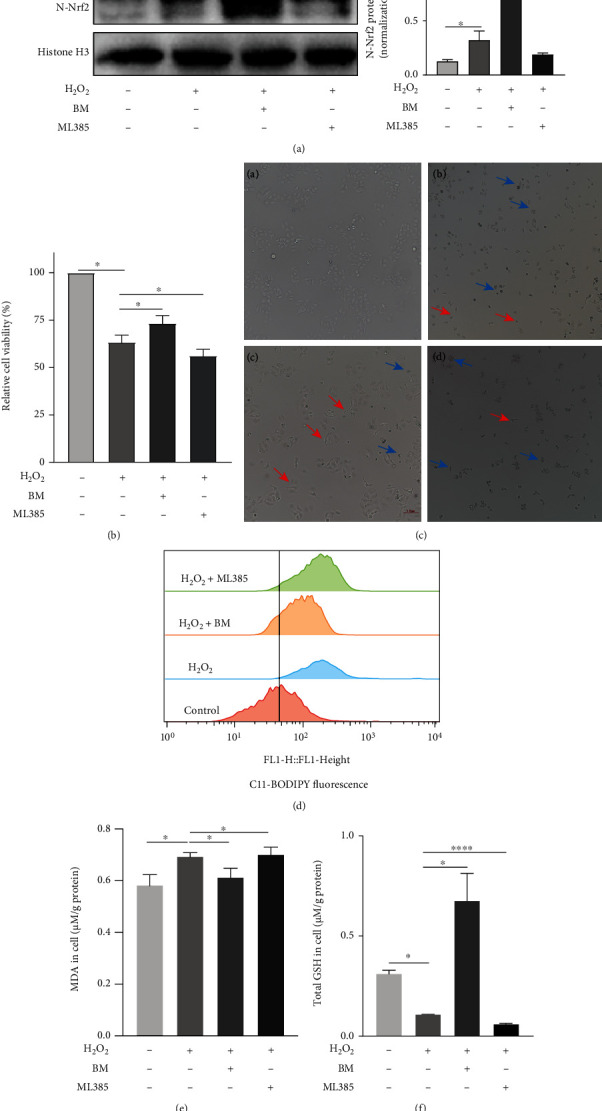
Nrf2 protected against H_2_O_2_-induced hepatocytes injury via inhibiting ferroptosis. (a) The results of western blot suggested the successful activation or inhibition of Nrf2 in vitro (*n* = 3). (b) BM treatment significantly increased the cell viability of L02 cells with H_2_O_2_ exposure, while ML385 significantly decreased L02 cell viability treated with H_2_O_2_ (*n* = 4). (c) BM treatment reduced the number of cells showing ferroptosis-specific morphologic phenotype. The blue and red arrows indicate shrunken and balloon phenotype, respectively. Original magnification ×200. (d) Flow cytometry was used to detect the content of cellular lipid peroxide after treating cells with 5 *μ*M BODIPY® 581/591 C11 (*n* = 3). (e and f) Cellular GSH and MDA content were measured. ^∗^*p* < 0.05, ^∗∗∗∗^*p* < 0.0001. BM = Bardoxolone Methyl; GSH = glutathione; MDA = malondialdehyde; Nrf2 = nuclear factor erythroid 2-related factor.

To explore effects of Nrf2 on H_2_O_2_-induced cell injury, BM (0.2 *μ*M) and ML385 (10 *μ*M) were applied to activate or inhibit Nrf2. As shown in Figure 8(a), nuclear expression of Nrf2 was increased upon BM treatment whereas decreased upon ML385 treatment, suggesting that Nrf2 was activated or inactivated, respectively. The mRNA (Supplemental Figure 1) and protein expression (Supplemental Figure 2) of HO-1 and NQO1 changed accordingly in response to activation or inhibition of Nrf2, respectively. BM treatment improved cell viability and morphologic features, while ML385 treatment had opposite effects (Figures 8(b) and 8(c)). These results indicated that Nrf2 might exert a protective effect on H_2_O_2_-induced cell damage. Effects of Nrf2 on H_2_O_2_-induced ferroptosis were investigated in L02 cells. BM treatment decreased lipid peroxidation during ACLF, as evidenced by reduced level of lipid ROS in the BM treatment group relative to the ACLF group (Figure 8(d)). In contrast, ML385 treatment augmented the accumulation of lipid ROS. Likewise, ML385 treatment aggravated the increase in MDA whereas decrease in GSH in L02 cells (Figures 8(e) and 8(f)). Taken together, Nrf2 could protect L02 cells from H_2_O_2_-induced ferroptosis.

## 4. Discussion

In this study, ferroptosis has been identified to participate in pathogenesis of ACLF, while inhibition of ferroptosis through activating Nrf2-mediated pathway is a potential strategy to prevent ACLF progression. Characterized by a heterogeneous and intertwined pathophysiological process and high short-term mortality, ACLF constitutes a significant threat to public health worldwide without clinically effective treatments [32]. Unveiling ACLF pathogenesis and seeking effective therapeutic targets would be a focus of clinical and basic research. Here, through establishing ACLF models by treating mice with CCl4, LPS, and D-Gal along with a hepatocyte injury model, for the first time we demonstrate participation of ferroptosis in ACLF pathogenesis as evidenced by the following: (1) ACLF livers exhibit key features of ferroptosis including lipid peroxidation ^6, 23^ (increase in MDA content whereas decrease in GSH and NADPH in ACLF models), upregulation of PTGS2, and presence of ferroptosis-specific mitochondrial morphology; (2) activation of ferroptosis exacerbates lipid peroxidation and leads to more severe liver damage, while inhibiting ferroptosis with ferroptosis inhibitor Fer-1 [8] largely abrogates injury and restores liver damage.

Ferroptosis is an iron-dependent nonapoptotic form of cell death resulting from excessive iron accumulation and lipid peroxidation. Massive iron accumulation, increased lipid peroxidation, and deficiency in cellular antioxidation are recognized as main pathological pillars during ferroptotic cascade [33]. Recently, accumulating evidence has indicated involvement of ferroptosis in liver diseases. Ferroptosis may contribute to liver injury and promote disease progression in acute or chronic liver diseases, such as ischemia/reperfusion-related injury and nonalcoholic fatty liver disease [8, 24]. Despite evidence that iron overload and increased oxidative stress have been revealed in ACLF patients, especially those with multiorgan failure [13, 14, 34, 35], no studies have investigated association between ferroptosis and ACLF, representing a gap in knowledge. For the first time, our current study has demonstrated that ferroptosis is responsible for aggravated liver damage in ACLF. Thus, inhibiting ferroptosis alleviates the severity of ACLF, providing novel therapeutic cues based on ferroptosis.

Nrf2 is a stress-inducible transcription factor that elicits defense to protect cells from oxidative injury through regulation of a host of defensive and detoxification genes [36] involved in iron metabolism, glutathione synthesis, and metabolism of reactive intermediates [37, 38]. Notably, most of its target genes are critical for ferroptosis. In addition, antiferroptosis mediators including glutathione peroxidase 4 (Gpx4) [39], Ferroptosis Suppressor Protein 1 (FSP1) [40], and system Xc^−^ [41] are all target genes of Nrf2. Thus, Nrf2-mediated antioxidant defense is integral in mitigating lipid peroxidation and ferroptosis prevention. For pathologic conditions where ferroptosis functions as a detrimental factor in disease development, activation of Nrf2 may play a beneficial role in attenuating damage through removing overwhelmed lipid peroxidation and unrelenting cell death [19]. For example, ferroptosis was activated after seawater drowning, and repression of ferroptosis relieved lung damage [30]. Further mechanical studies using inhibitor/inducer of Nrf2 suggested that activation of Nrf2 improved the severity of acute lung injury via decreasing lipid peroxidation [30].

In the present study, nuclear expression of Nrf2 was upregulated in ACLF model and hepatocyte injury model, implying that Nrf2 may activate adaptively to combat increased lipid peroxidation. The end products of lipid peroxidation themselves, including MDA and 4-hydroxynonenal (4-HNE), are potent initiators of lipid peroxidation [42]. As such, pharmacologically activated Nrf2 might have a synergistic effect with originally activated Nrf2 to control rapidly increased lipid peroxidation. To test this hypothesis, inhibitor/inducer of Nrf2 (ML385 and BM) was applied. As expected, BM treatment induced Nrf2 expression, improved cell viability, reduced MDA and lipid ROS, and restored depleted GSH in L02 cells after H_2_O_2_ exposure. As depicted in liver histopathology, BM treatment reduced histological lesions in ACLF mice. Mounting evidence has revealed the important role of Nrf2 in ameliorating inflammatory responses [43–46]. Consistently proinflammatory cytokines TNF-*α* and IL-6 were decreased in BM-treated mice compared with ACLF mice, suggesting a reduced inflammatory response. In addition, increased GSH and NADPH whereas decreased MDA and PTGS2 mRNA expression suggested that BM mitigated ferroptosis in vivo. Inhibitory effects of Nrf2 on ferroptosis were verified by using Nrf2 inhibitor ML385. As expected, ML385 exerted opposite effects to BM. Overall, Nrf2 could attenuate liver damage via inhibiting ferroptosis and inflammatory response in ACLF. Although inhibiting ferroptosis may have an anti-inflammatory effect [47], our current study observed no significant decrease in TNF-*α* and IL-6 in mice treated with ferroptosis inhibitor compared with ACLF mice. More research is needed to determine potential roles of ferroptosis in inflammatory response in ACLF. Previous studies indicated that BM might increase serum aminotransferase levels through inducing expression of aminotransferases as an on-target effect [48], which may partly explain why ALT and AST levels were not decreased after BM treatment in mice (Figure 3(f)).

Despite novel findings, some limitations of this study should be kept in mind. Bardoxolone Methyl is a potent noncytotoxic activator of Nfr2 and has been tested in clinical trials for chronic kidney and malignant diseases [49, 50]. However, because of multifunctional property such as an inhibitor nuclear factor-*κ*B [51], precise net effects of Nrf2 on ferroptosis in ACLF should be examined via genetic manipulation of Nrf2. In addition, upstream regulators of Nrf2 in ACLF need investigation.

## 5. Conclusions

In summary, our present study provides the first evidence that ferroptosis is a major RCD in ACLF. Inhibiting ferroptosis by Nrf2 could alleviate liver damage and prevent hepatocyte death. This study implies that targeting Nrf2-mediated ferroptosis may be a promising therapeutic approach in treating ACLF. Bardoxolone Methyl may be a potential new treatment option for ACLF patients.

## Figures and Tables

**Table 1:** Primers used in this study.

	Gene	Forward	Reverse
Human	PTGS2	5′-TGAGCATCTACGGTTTGCTG-3′	5′-TGCTTGTCTGGAACAACTGC-3′
	GAPDH	5′-TGTCATGGCAGAAGTACCTG-3′	5′-GTTAACTGGGGAGCCTGCTC-3′
	HO-1	5′-CCAGGCAGAGAATGCTGAGTTC-3′	5′-AAGACTGGGCTCTCCTTGTTGC-3′
	NQO-1	5′-CCTGCCATTCTGAAAGGCTGGT-3′	5′-GTGGTGATGGAAAGCACTGCCT-3′

Mouse	PTGS2	5′-CTGCGCCTTTTCAAGGATGG-3′	5′-GGGGATACACCTCTCCACCA-3′
	GAPDH	5′-CAAAGCAAAGATGCTCCACA-3′	5′-ATCGCATGAACCTTGTTTCC-3′

## Data Availability

The data that support the findings of this study are available from the corresponding author upon reasonable request.

## Supplementary Materials

Supplementary 1
Figure 1: the mRNA expression of Nrf2 target genes. A. The mRNA expression of NQO1 was upregulated in ACLF patients relative to healthy controls. (HC *n* = 3, ACLF *n* = 5). B. The mRNA expression of HO-1 was increased after H_2_O_2_ exposure, and BM treatment augmented its expression, while ML385 inhibited its expression (*n* = 3).

Supplementary 2
Figure 2: the protein expression of Nrf2 target genes. A. The protein expressions of NQO1 and HO-1 were increased in response to H_2_O_2_ and Erastin treatment, while Fer-1 treatment reversed the effect of H_2_O_2_ (*n* = 3). B. The protein expressions of HO-1 and NQO1 were increased after H_2_O_2_ exposure, and BM treatment augmented its expression, while ML385 inhibited its expression (*n* = 3). ACLF, acute-on-chronic liver failure; BM, Bardoxolone Methyl; E, Erastin; Fer-1, ferrostatin-1; HO-1, heme oxygenase-1; NQO1, NAD(P) H quinone dehydrogenase, quinone 1.
