# Supplementary figures and images for: Nrf2-Mediated Ferroptosis Inhibition Exerts a Protective Effect on Acute-on-Chronic Liver Failure
Source: Oxid Med Cell Longev. 2022 Apr 16;2022:4505513. doi: 10.1155/2022/4505513 (PMC9036161; doi:10.1155/2022/4505513)

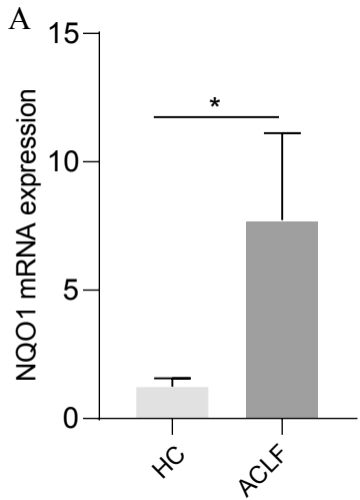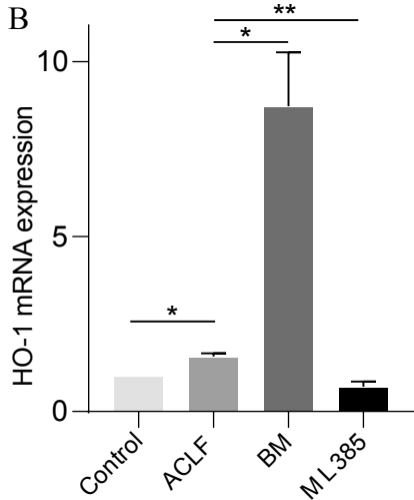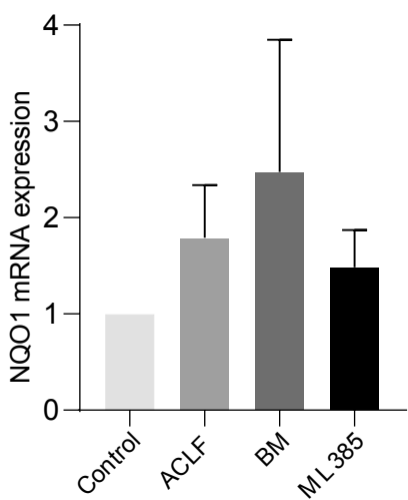

Supplement: Supplementary 1 — Figure 1: the mRNA expression of Nrf2 target genes. A. The mRNA expression of NQO1 was upregulated in ACLF patients relative to healthy controls. (HC n = 3, ACLF n = 5). B. The mRNA expression of HO-1 was increased after H2O2 exposure, and BM treatment augmented its expression, while ML385 inhibited its expression (n = 3). [file 4505513.f1.pdf]

A

NQO1

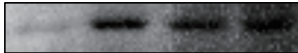

HO-1

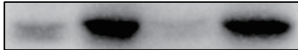

GAPDH

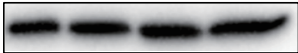

|                               |   |   |   |   |
|-------------------------------|---|---|---|---|
| H <sub>2</sub> O <sub>2</sub> | - | + | + | - |
| Fer-1                         | - | - | + | - |
| E                             | - | - | - | + |

B

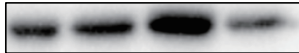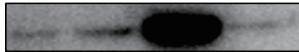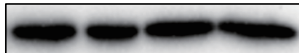

|                               |   |   |   |   |
|-------------------------------|---|---|---|---|
| H <sub>2</sub> O <sub>2</sub> | - | + | + | + |
| BM                            | - | - | + | - |
| ML385                         | - | - | - | + |

Supplement: Supplementary 2 — Figure 2: the protein expression of Nrf2 target genes. A. The protein expressions of NQO1 and HO-1 were increased in response to H2O2 and Erastin treatment, while Fer-1 treatment reversed the effect of H2O2 (n = 3). B. The protein expressions of HO-1 and NQO1 were increased after H2O2 exposure, and BM treatment augmented its expression, while ML385 inhibited its expression (n = 3). ACLF, acute-on-chronic liver failure; BM, Bardoxolone Methyl; E, Erastin; Fer-1, ferrostatin-1; HO-1, heme oxygenase-1; NQO1, NAD(P) H quinone dehydrogenase, quinone 1. [file 4505513.f2.pdf]
